# Supplementary material for: Designing Coulombic Contact Interactions between Polarizable Particles through Asymmetry
Source: J Chem Theory Comput. 2026 Aug 24;22(19):9954–62. doi: 10.1021/acs.jctc.6c00946 (PMC13671168; doi:10.1021/acs.jctc.6c00946)
Supplement: Supplementary file 1 [file ct6c00946_si_001.pdf]

Supporting Information for "Designing Coulombic Contact Interactions between Polarizable Particles through Asymmetry"

Yanyu Duan[†] and Zecheng Gan[*,†,‡]

[†] *Thrust of Advanced Materials, and Guangzhou Municipal Key Laboratory of Materials Informatics, The Hong Kong University of Science and Technology (Guangzhou), Guangzhou 511453, China*

[‡] *Department of Mathematics, The Hong Kong University of Science and Technology, Clear Water Bay, Kowloon, Hong Kong SAR, 999077, China*

E-mail: zechenggan@ust.hk

Contact energy validation

This section validates the leading-order contact-energy expression used to generate the analytical design rules in the main text. The system parameters, calculated contact energies, benchmark reference values, and relative errors are listed in Table 1. The relative contact-energy error is defined as $\delta_E = |E_{\mathrm{ele}} - E_{\mathrm{Ref}}|/|E_{\mathrm{Ref}}| \times 100\%$.

Table 1: Contact-energy validation for a pair of identical dielectric spheres with charges ($Q_1 = Q_2 = Q$), dielectric constants ($\epsilon_1 = \epsilon_2 = \epsilon_{\rm in}$), and radii ($a_1 = a_2 = a$), suspended in a homogeneous medium with permittivity $\epsilon_{\rm out}$. The dielectric contrast is defined as $k = (\epsilon_{\rm in} - \epsilon_{\rm out})/(\epsilon_{\rm in} + \epsilon_{\rm out})$. Both $E_{\rm Ref}$ and $E_{\rm ele}$ are contact energies: $E_{\rm Ref}$ denotes reference values from the literature,[1] and $E_{\rm ele}$ denotes the leading-order analytical result used in this work. Both energies are expressed in units of $\frac{Q^2}{4\pi\varepsilon_0\epsilon_{\rm out}a}$.

k	$E_{\rm Ref}$	$E_{\rm ele}$	δ_E
-0.960	0.563	0.544	3.375%
-0.871	0.556	0.541	2.698%
-0.775	0.549	0.537	2.186%
-0.678	0.542	0.533	1.661%
-0.581	0.535	0.529	1.121%
-0.488	0.529	0.525	0.756%
-0.391	0.523	0.521	0.382%
-0.295	0.516	0.516	0
-0.196	0.511	0.511	0
-0.099	0.505	0.505	0
0	0.5	0.5	0
0.042	0.496	0.497	0.202%
0.188	0.487	0.488	0.205%
0.315	0.480	0.480	0
0.476	0.471	0.468	0.637%
0.639	0.461	0.454	1.518%
0.784	0.452	0.440	2.655%
0.960	0.442	0.421	4.751%

Analysis for contact energy: the role of asymmetries

Two-sphere systems with charge asymmetry

We first isolate charge asymmetry by setting the dielectric constants and radii to be symmetric, i.e., $\epsilon_1 = \epsilon_2 = \epsilon_{\text{in}}$ ($k_1 = k_2 = k$), and $a_1 = a_2 = a$ ($t_1 = t_2 = t = \frac{a}{2a} = \frac{1}{2}$). Therefore, the contact energy expression in the main text reduces to

$$E_{\text{ele}} = \frac{Q_1 Q_2}{4\pi\varepsilon_0\epsilon_{\text{out}}(2a)} \left\{ 1 - \frac{k}{4}\left(\frac{Q_1}{Q_2} + \frac{Q_2}{Q_1}\right)\left[\frac{4}{3} - (1-k)\sum_{n=0}^{\infty}\frac{4^{-n}}{2n+1-k}\right]\right\} . \tag{1}$$

Figs. 1(a) and 1(b) illustrate the impact of charge asymmetry on the contact energy of like-charged and oppositely charged spheres, respectively. In each scenario, we plot the contact energy normalized by the pure Coulomb energy, $E_{\text{ele}}/E_{\text{Coul}}$, versus $(Q_1/Q_2+Q_2/Q_1)$, with varying dielectric contrast values $k \in (-1, 1)$. The dimensionless parameter $(Q_1/Q_2 + Q_2/Q_1)$, which appears in Eq. (1), characterizes the strength of charge asymmetry: for like-charged spheres, it reaches its minimum value of 2 when $Q_1 = Q_2$; for oppositely charged spheres, its magnitude increases as the charge asymmetry increases.

Figs. 1(a) and 1(b) show that, in the absence of dielectric contrast ($k = 0$), the contact energy is equal to the pure Coulomb energy. When $k \neq 0$, the contact energy deviates from the Coulomb value as the charge asymmetry or dielectric mismatch increases. For conductor-like spheres ($k > 0$), the normalized contact energy is lower than the Coulomb value, indicating weakened like-charge repulsion or enhanced opposite-charge attraction. For insulator-like spheres ($k < 0$), the normalized contact energy is higher than the Coulomb value, indicating enhanced like-charge repulsion or weakened opposite-charge attraction. These trends are consistent with earlier studies of polarization-induced like-charge attraction and opposite-charge repulsion.[2,3]

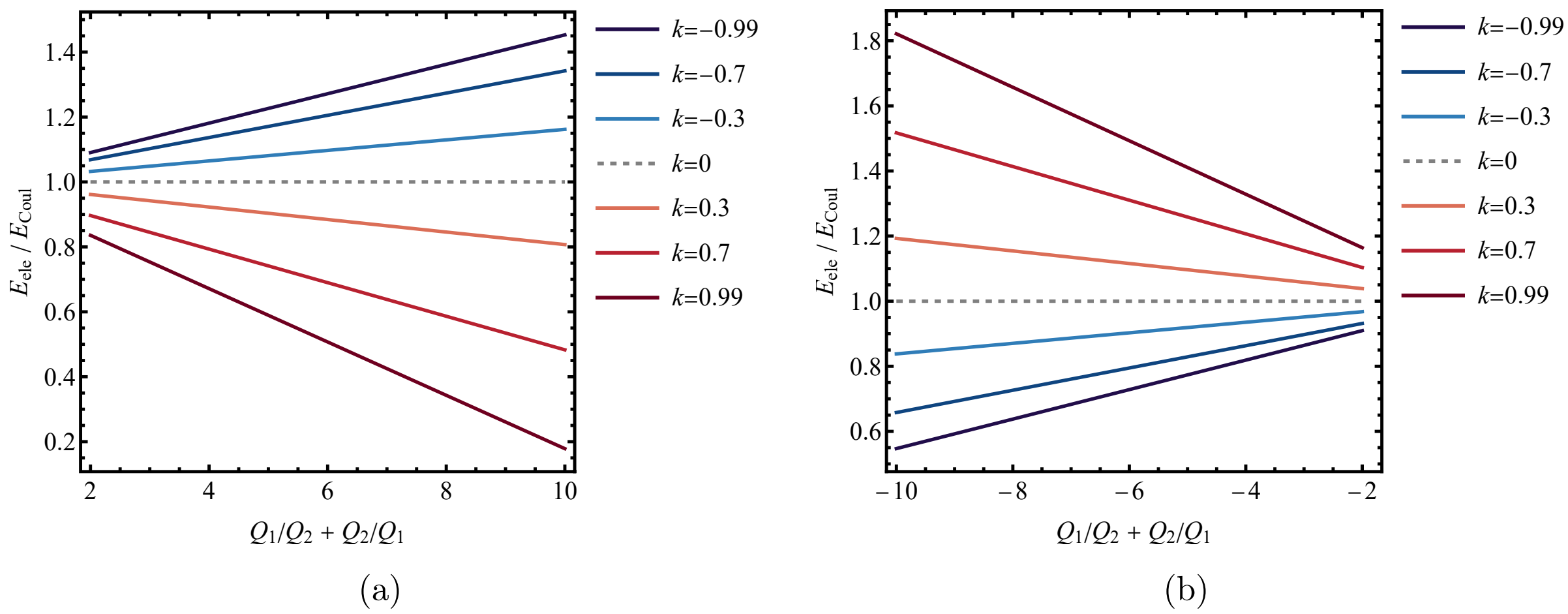

Figure 1: Influence of charge asymmetry $\left(\frac{Q_1}{Q_2} + \frac{Q_2}{Q_1}\right)$ on the contact energy of two-sphere systems. (a) Like-charged spheres ($Q_1Q_2 > 0$) and (b) oppositely charged spheres ($Q_1Q_2 < 0$). In each scenario, the normalized contact energy $E_{\text{ele}}/E_{\text{Coul}}$ is plotted versus $(Q_1/Q_2 + Q_2/Q_1)$ for different dielectric contrasts k.

Two-sphere systems with size asymmetry

We next isolate size asymmetry by considering a pair of spheres with radii a_1 and a_2, while setting $Q_1 = Q_2 = Q$ and $k_1 = k_2 = k$. For contacting spheres, the contact ratios satisfy $t_1 + t_2 = 1$, where $t_1 = a_1/(a_1 + a_2)$ and $t_2 = a_2/(a_1 + a_2)$. Hence, by substituting $t_2 = 1 - t_1$, the contact energy expression in the main text becomes

$$E_{\text{ele}} = \frac{Q^2}{4\pi\varepsilon_0\epsilon_{\text{out}}(a_1 + a_2)} \left\{ 1 - \frac{kt_1}{2}\left[\frac{1}{1-t_1^2} - (1-k)\sum_{n=0}^{\infty}\frac{t_1^{2n}}{2n+1-k}\right] - \frac{k(1-t_1)}{2}\left[\frac{1}{t_1(2-t_1)} - (1-k)\sum_{n=0}^{\infty}\frac{(1-t_1)^{2n}}{2n+1-k}\right]\right\}. \tag{2}$$

In Fig. 2, we plot the contact energy (normalized by the pure Coulomb energy E_{Coul}) versus t_1, with varying dielectric contrast $k \in (-1, 1)$. Fig. 2 shows that, when the two spheres are equal sized ($t_1 = t_2 = 0.5$), the contact energy reaches an extremum for each dielectric contrast k. As the size asymmetry increases (t_1 approaches either 0 or 1), the contact energy deviates increasingly from the pure Coulomb energy E_{Coul}. Under extreme dielectric contrast and size asymmetry, the contact energy can become several times larger

than the pure Coulomb value or even reverse sign because of strong polarization. As in Fig. 1, conductor-like spheres ($k > 0$) weaken the normalized contact energy, whereas insulator-like spheres ($k < 0$) enhance it.

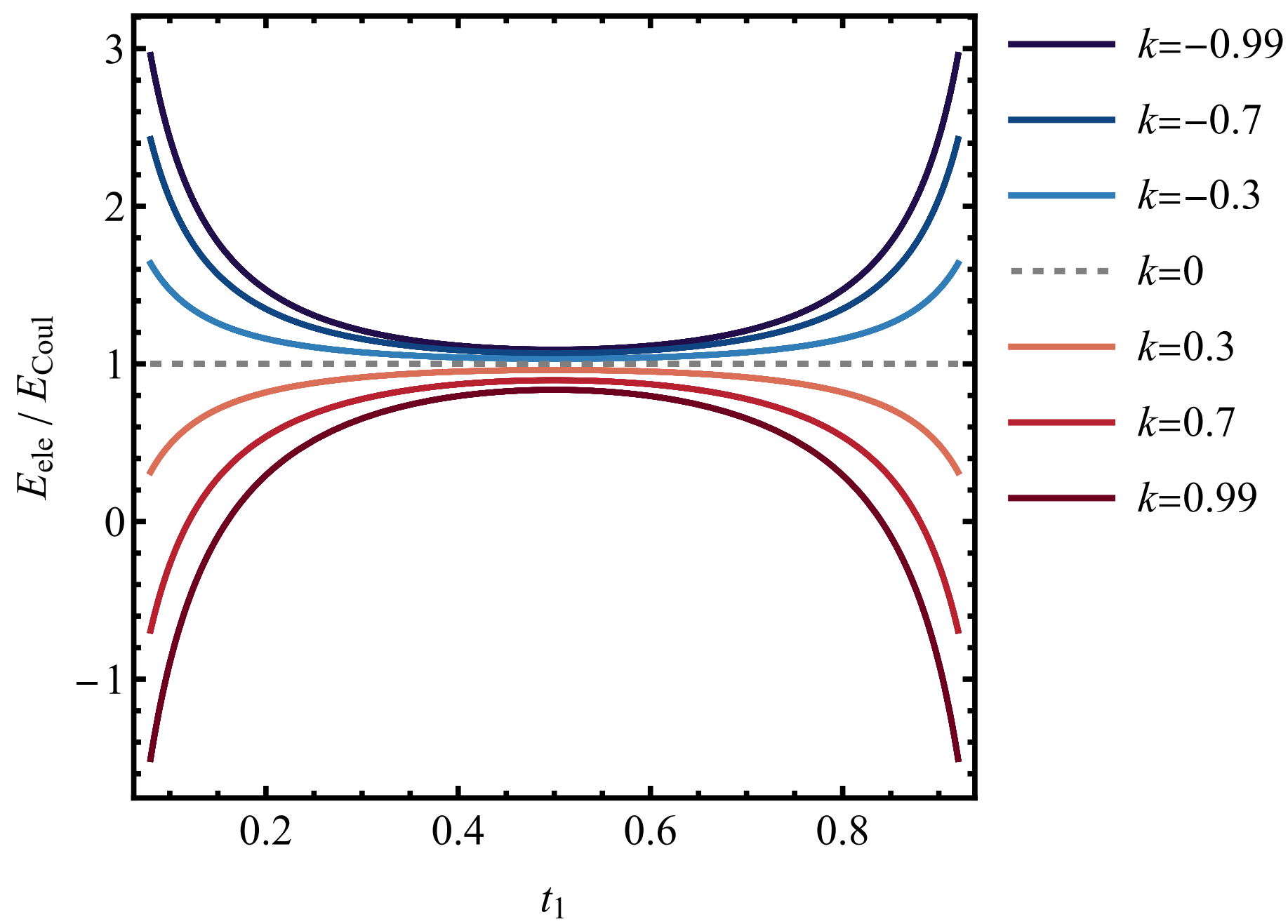

Figure 2: Influence of size asymmetry ($t_1 \neq t_2$) on the contact energy of two-sphere systems. The normalized contact energy $E_{\text{ele}}/E_{\text{Coul}}$ is plotted versus t_1 for different dielectric contrasts $k \in (-1, 1)$.

Validation of the pure Coulomb contact force

Tables 2 and 3 document contact-force validation for representative parameter sets predicted by the analytical design rules and marked in the main-text figures. Table 2 collects the validation data for two-sphere systems with charge and dielectric asymmetries, whereas Table 3 collects the data for systems with size and dielectric asymmetries. For each set of four points with a fixed charge or size asymmetry, the RMS contact-force deviation is $\delta_F = \sqrt{\frac{1}{4}\sum_{i=1}^{4}(F^i_{\text{Simul}} - F_{\text{Coul}})^2}/|F_{\text{Coul}}| \times 100\%$.

Table 2: Contact-force validation for two equal-sized dielectric spheres in vacuum ($\epsilon_{\text{out}} = 1$). In the simulations, $Q_2 = 1\mu C$ and $a_1 = a_2 = a = 1\mu m$, so the force is reported in units of $\frac{1}{4\pi\varepsilon_0}\frac{[C]^2}{[m]^2}$. F_{Coul} and F_{Simul} denote the pure Coulomb force and the force calculated using the Hybrid Method,[4] respectively.

$\frac{Q_1}{Q_2}$	k_1	k_2	ϵ_1	ϵ_2	Q_2	F_{Coul}	F_{Simul}	δ_F
0.3	0.065	-0.932	1.139	0.040	1	0.075	0.073	1.493%
	0.02	-0.239	1.041	0.614	1		0.075	
	-0.065	0.587	0.878	3.843	1		0.074	
	-0.02	0.208	0.961	1.525	1		0.075	
0.7	0.09	-0.199	1.198	0.668	1	0.175	0.174	2.617%
	0.2	-0.495	1.5	0.338	1		0.168	
	-0.09	0.170	0.835	1.410	1		0.178	
	-0.2	0.347	0.667	2.063	1		0.170	
1	0.25	-0.292	1.667	0.548	1	0.25	0.242	2.020%
	0.1	-0.106	1.222	0.808	1		0.249	
	-0.25	0.218	0.6	1.558	1		0.244	
	-0.1	0.095	0.818	1.210	1		0.249	
1.5	0.12	-0.056	1.273	0.894	1	0.375	0.374	1.347%
	0.3	-0.153	1.857	0.735	1		0.367	
	-0.12	0.051	0.786	1.107	1		0.374	
	-0.3	0.119	0.538	1.270	1		0.369	
2.5	0.45	-0.085	2.626	0.843	1	0.625	0.614	1.067%
	0.2	-0.034	1.5	0.934	1		0.623	
	-0.45	0.063	0.379	1.134	1		0.618	
	-0.2	0.030	0.667	1.062	1		0.623	

Table 3: Contact-force validation for two unequal-sized dielectric spheres in vacuum ($\epsilon_{\text{out}} = 1$). In the simulations, $Q_1 = Q_2 = 1\mu C$, and a_1 and a_2 are measured in units of μm, so the force is reported in units of $\frac{1}{4\pi\varepsilon_0}\frac{[C]^2}{[m]^2}$. F_{Coul} and F_{Simul} denote the pure Coulomb force and the force calculated using the Hybrid Method,[4] respectively.

t_1	t_2	a_1	a_2	k_1	k_2	ϵ_1	ϵ_2	F_{Coul}	F_{Simul}	δ_F
0.38	0.62	0.613	1	0.4	-0.073	2.333	0.864	0.384	0.383	0.813%
				0.15	-0.025	1.353	0.951		0.385	
				-0.4	0.055	0.429	1.116		0.378	
				-0.15	0.022	0.739	1.045		0.383	
0.45	0.55	0.818	1	0.25	-0.131	1.667	0.768	0.303	0.299	1.082%
				0.1	-0.049	1.222	0.907		0.302	
				-0.25	0.106	0.6	1.237		0.298	
				-0.1	0.045	0.818	1.094		0.302	
0.5	0.5	1	1	0.15	-0.164	1.353	0.718	0.25	0.247	0.775%
				0.08	-0.084	1.174	0.845		0.249	
				-0.15	0.138	0.739	1.320		0.248	
				-0.08	0.076	0.852	1.165		0.249	
0.54	0.46	1	0.852	0.15	-0.314	1.353	0.522	0.292	0.285	1.579%
				0.1	-0.200	1.222	0.667		0.288	
				-0.15	0.245	0.739	1.649		0.288	
				-0.1	0.170	0.818	1.410		0.290	
0.66	0.34	1	0.515	0.05	-0.782	1.105	0.122	0.436	0.424	1.455%
				0.02	-0.271	1.041	0.574		0.432	
				-0.05	0.513	0.905	3.107		0.436	
				-0.02	0.229	0.961	1.594		0.437	

Completeness and reproducibility of MD simulations

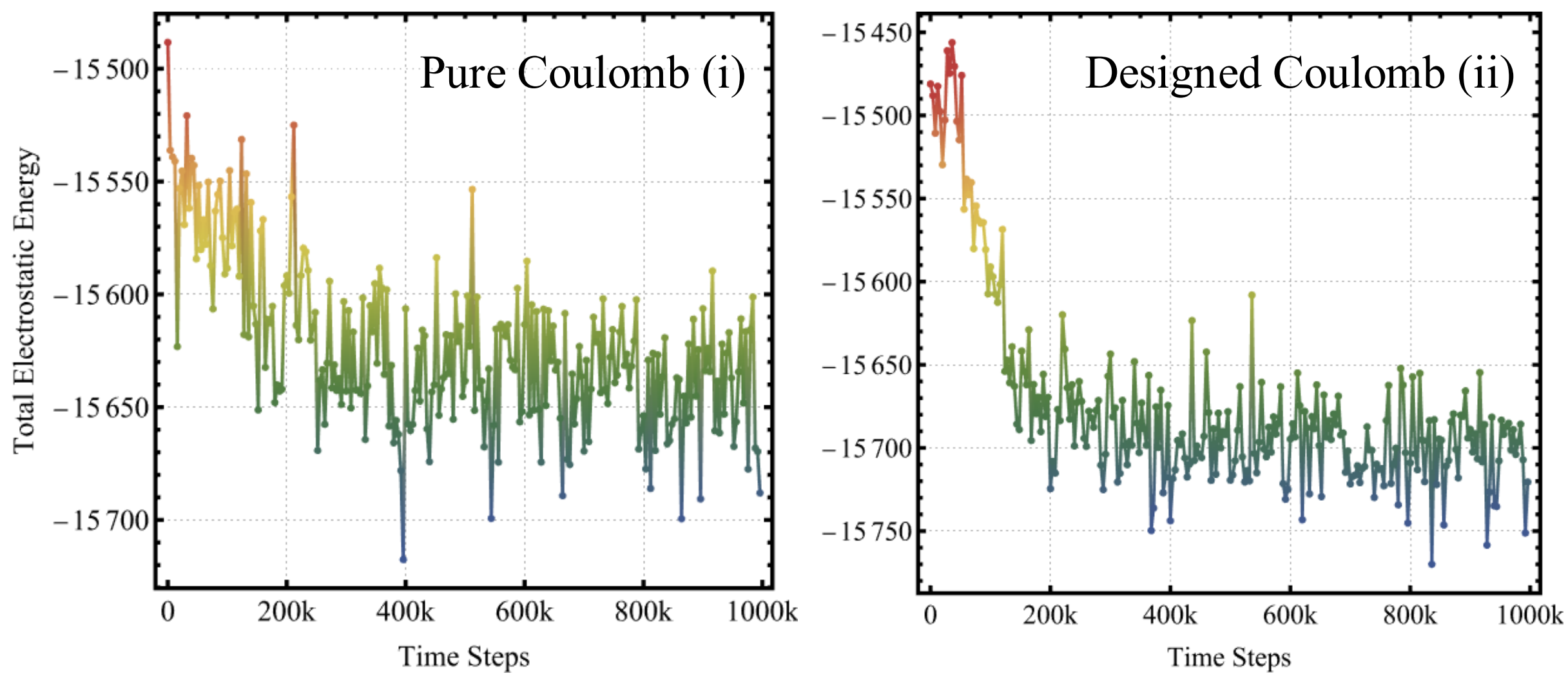

(a)

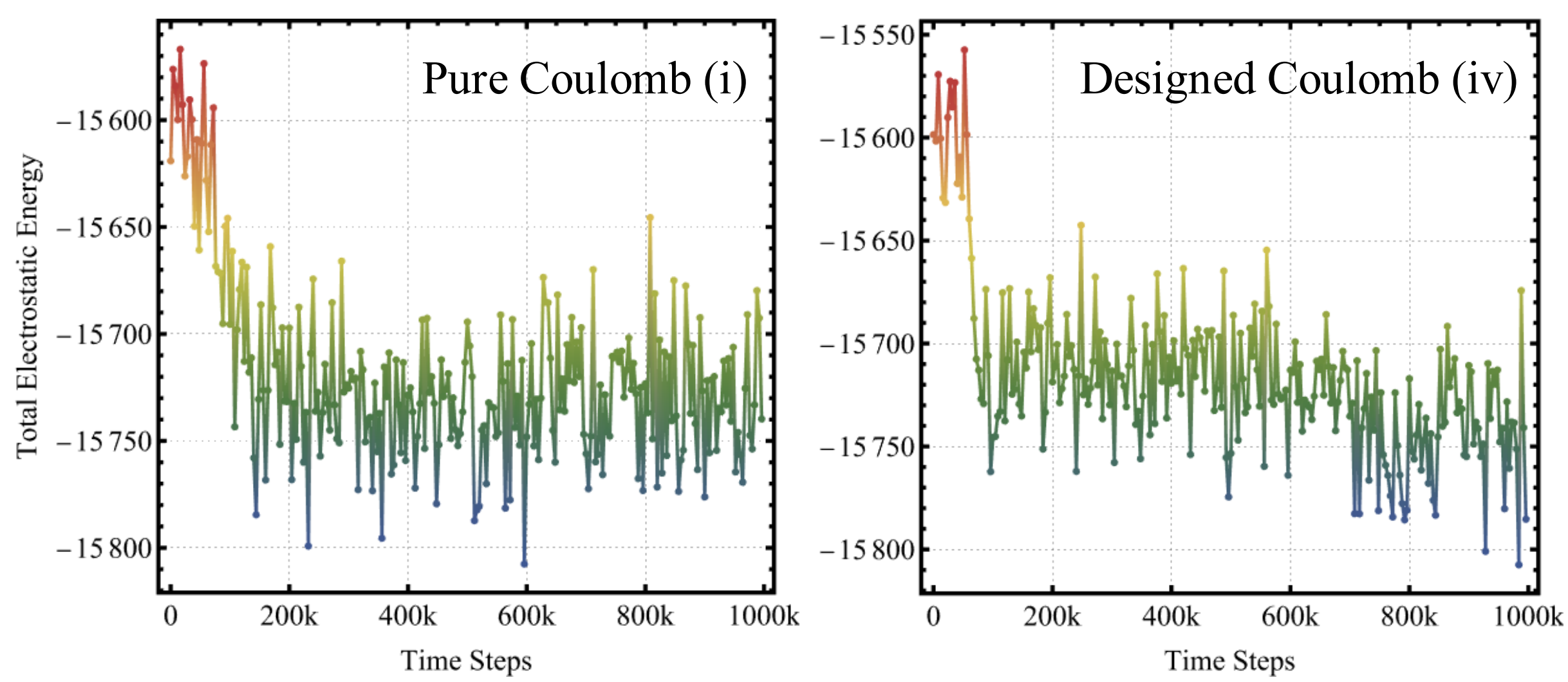

(b)

Figure 3: Time evolution of the total electrostatic energy during MD simulations of oppositely charged spheres ($N = 200$). (a) Equal-sized systems, where Pure Coulomb (i) and Designed Coulomb (ii) correspond to Fig. 5(a) of the manuscript. (b) Unequal-sized systems, where Pure Coulomb (i) and Designed Coulomb (iv) correspond to Fig. 5(b) of the manuscript. The first 2×10^5 steps cover the initial energy relaxation and were discarded before production averages were calculated.

References

(1) Lian, H.; Qin, J. Polarization energy of two charged dielectric spheres in close contact. *Mol. Syst. Des. Eng.* **2018**, *3*, 197–203.

(2) Duan, Y.; Gan, Z. Quantitative theory for critical conditions of like-charge attraction between polarizable spheres. *J. Chem. Theory Comput.* **2025**, *21*, 2822–2828.

(3) Duan, Y.; Gan, Z.; Chan, H.-K. Mechanisms of electrostatic interactions between two charged dielectric spheres inside a polarizable medium: An effective-dipole analysis. *Soft Matter* **2025**, *21*, 1860–1872.

(4) Gan, Z.; Wang, Z.; Jiang, S.; Xu, Z.; Luijten, E. Efficient dynamic simulations of charged dielectric colloids through a novel hybrid method. *J. Chem. Phys.* **2019**, *151*, 024112.
